# The Clinical Significance of IDH Mutations in Essential Thrombocythemia and Primary Myelofibrosis

**DOI:** 10.14740/jocmr2405w

**Published:** 2015-12-03

**Authors:** Ipek Yonal-Hindilerden, Aynur Daglar-Aday, Fehmi Hindilerden, Basak Akadam-Teker, Ceylan Yilmaz, Meliha Nalcaci, Akif Selim Yavuz, Deniz Sargin

**Affiliations:** a Division of Hematology, Department of Internal Medicine, Istanbul University Istanbul Medical Faculty, Istanbul, Turkey; b Hematology Clinic, Istanbul Bakirkoy Sadi Konuk Training and Research Hospital, Istanbul, Turkey; c Division of Hematology, Department of Internal Medicine, Medipol University, Istanbul, Turkey

**Keywords:** Philadelphia-negative myeloproliferative neoplasms, IDH1, IDH2

## Abstract

**Background:**

Limited data exist regarding impact of IDH mutations in Philadelphia-negative myeloproliferative neoplasms (Ph-negative MPNs). Prognostic significance of IDH mutations was asessed in 184 Ph-negative MPN patients - 107 essential thrombocythemia (ET) and 77 primary myelofibrosis (PMF).

**Methods:**

High-resolution melting (HRM) analysis was used to detect IDH1 and IDH2 mutations.

**Results:**

PMF and ET patients showed no significant difference for prevalence of IDH mutations. Mutant IDH (IDH1 or IDH2) was documented in five of PMF (6.5%) and two of ET patients (1.9%). IDH mutations in ET patients included one IDH1 R132C and one IDH2 R140Q. Of the five IDH-mutated PMF patients, four (80%) displayed IDH1 (three IDH1 R132C and one IDH1 R132S) and one (20%) carried IDH2 (IDH2 R140Q) mutation. Sixty percent (three in five) of IDH-mutated PMF patients carried JAK2V617F with following allele burdens: 31-50%, 5-12.5% and 31-50%, respectively. Three of 77 PMF patients (3.9%) simultaneously harbored IDH and JAK2V617F mutations. IDH mutations in PMF showed a trend towards higher rate in females (100% and 52.8%, respectively). Bleeding complications were significantly higher in IDH-mutated PMF patients compared to IDH wild-type counterparts. Trend towards a lower prevalance of acetylsalicylic acid (ASA) use was present in IDH mutant PMF patients compared to wild-type counterparts (20% and 63.9%, respectively). Death rate was higher in IDH-mutated PMF patients compared to IDH wild-type PMF patients (60% and 15.3%). In univariate analysis, a significantly shorter leukemia-free survival (LFS) was observed in IDH-mutated PMF patients.

**Conclusions:**

We conclude that IDH mutations indicate a risk for leukemic transformation in PMF.

## Introduction

IDH1 (located on chromosome 2q33.3) and IDH2 (located on chromosome 15q26.1) encode two seperate different isocitrate dehydrogenase enzymes. These enzymes catalyze oxidative decarboxylation of isocitrate to α-ketoglutarate (α-KG). IDH mutations are located on exon 4 and involve a change of a single aminoacid in the arginine residue. IDH mutations affect three specific arginine residues: R132 (IDH1), R172 (the IDH1 R132 analogous residue on IDH2) and R140 (IDH2) [1]. Mutant IDH possesses catalytic activity to convert α-KG to 2-hydroxyglutarate (2-HG) [2-4]. The accumulation of 2-HG is thought to attribute to the oncogenic feature of mutant IDH [5].

Integrated genomic analysis identified recurrent mutations in the active site of IDH1 in 12% of glioblastoma multiforme patients [6]. Subsequently, another study showed the presence of mutated IDH1 (R132) and IDH2 (R172) genes in a majority of grade II or III gliomas and in secondary glioblastomas [7]. Also, genome-wide screening detected IDH1 mutation as a recurrent event in acute myeloid leukemia (AML) [8]. In the aforementioned study, IDH1 mutations (R132) were detected in 16 of 188 AML patients (9%), while IDH2 mutations were not detected [8]. In another study including 493 adult AML patients, IDH1 mutations (R132) were detected in 27 patients (6%) [9]. In both studies, IDH1 mutations were associated with normal cytogenetic status, trisomy 8 and NPM1 mutations [8, 9]. Also, IDH2 exon 4 mutations (R172, R140) were demostrated in AML patients [2, 3]. In general, IDH mutations did not seem to have an impact on survival in primary AML [2, 8, 9].

IDH1 and IDH2 mutations were also identified in chronic myeloid neoplasms, including MDS and myeloproliferative neoplasms (MPNs) [1, 10-19]. IDH mutations were detected at a frequency of 3-5% in MDS, 1-4% in MPN, 9% in MDS/MPN including CMML, 15% in AML transformed from MDS, 22% in AML transformed from MPN, 10% in AML transformed from MPN/MDS and 22% in high risk MDS associated with del(5q) [11-13, 20-23]. Contrary to AML, there is limited information on the prognostic significance of IDH mutations in chronic myeloid neoplasms [1, 11, 12, 17, 18]. In one study including 200 patients with chronic- and blast-phase MPNs, screening of IDH1 and IDH2 mutations determined that IDH mutations are relatively frequent in blast- but not chronic-phase MPN (21% and 4%, respectively). This suggested the possibility that IDH mutations in MPN represent early genetic events facilitating leukemic transformation [15]. Another study examining the impact of IDH1 and IDH2 mutations on phenotypic features and prognosis in 301 chronic-phase PMF patients implied that IDH mutations are independent risk factors for leukemic transformation in PMF and increase the leukemogenic risk in the copresence of JAK2V617F mutation [1]. The last comprehensive study which focused on the correlation of IDH mutations with prognosis in PMF enrolled 374 and 483 patients from the Mayo Clinic and the European Cohort, respectively [24]. IDH mutations showed correlation with shortened overall survival (OS) and leukemic transformation in the Mayo Clinic while in the European cohort, IDH1/IDH2 mutations predicted leukemic transformation but had no impact on OS [24].

In the current study, we set out to explore the prevalence and prognostic significance of IDH1 and IDH2 mutations in a relatively large cohort of Turkish MPN patients (n = 184). Clinical and hematologic parameters were also analyzed.

## Materials and Methods

### Patients

The study was approved by local ethics committee of Istanbul University Istanbul Medical Faculty and performed according to the principles of the Declaration of Helsinki. All participants provided informed written consent for study sample collection and permission for use in research. The study includes 184 patients with Ph-negative MPNs - 107 essential thrombocythemia (ET) and 77 primary myelofibrosis (PMF) patients - diagnosed according to the 2008 WHO criteria recruited between May 1995 and July 2013 [25]. Data obtained at study entry involved demographics, laboratuary features at diagnosis such as blood counts, lactate dehydrogenase (LDH) levels, and complications, such as bleeding and thrombosis. Medical history of red blood cell transfusion, medications, splenectomy and allogeneic hematopoietic stem cell transplantation (AHSCT) was recorded. Risk factors for cardiovascular diseases - diabetes mellitus, hypertension, cigarette smoking and dyslipidemia - were questioned. Mild and massive splenomegalies were defined as enlarged spleen with a longitudinal diameter of ≥ 130 mm up to 160 mm and ≥ 160 mm on ultrasound, respectively. Risk stratification of PMF patients was based on Dynamic International Prognostic Scoring System (DIPSS) plus [26]. Karyotypes of bone marrow or peripheral blood cells were described according to International System for Human Cytogenetic Nomenclature (ISCN) guidelines [27]. Unfavorable karyotypes include complex karyotype or one or two abnormalities including +8, -7/7q-, i(17q), inv(3), -5/5q-, 12p^-^, or 11q23 rearrangement [28]. Cytogenetic abnormalities other than the aferomentioned aberrations were defined as favorable karyotype abnormalities. Patients with no cytogenetic abnormalities were classified to have normal karyotype. OS was calculated as months from the date of diagnosis to the date of death or last contact. Leukemia-free survival (LFS) was calculated as months from the time of diagnosis to the time of leukemic transformation.

### Genotyping for IDH1 mutation (R132) and IDH2 mutation (R140 and R172)

IDH1/2 mutations were assessed at the Molecular Hematology Laboratory of Istanbul University. Genomic DNA was extracted from peripheral blood granulocytes using a high pure polymerase chain reaction (PCR) template preparation kit (Roche Diagnostic, Mannheim, Germany). The concentration of DNA was measured using a Nano-Drop-2000 spectrophotometer (Thermo Scientific, Wilmington, DE, USA).

**Figure 1:**
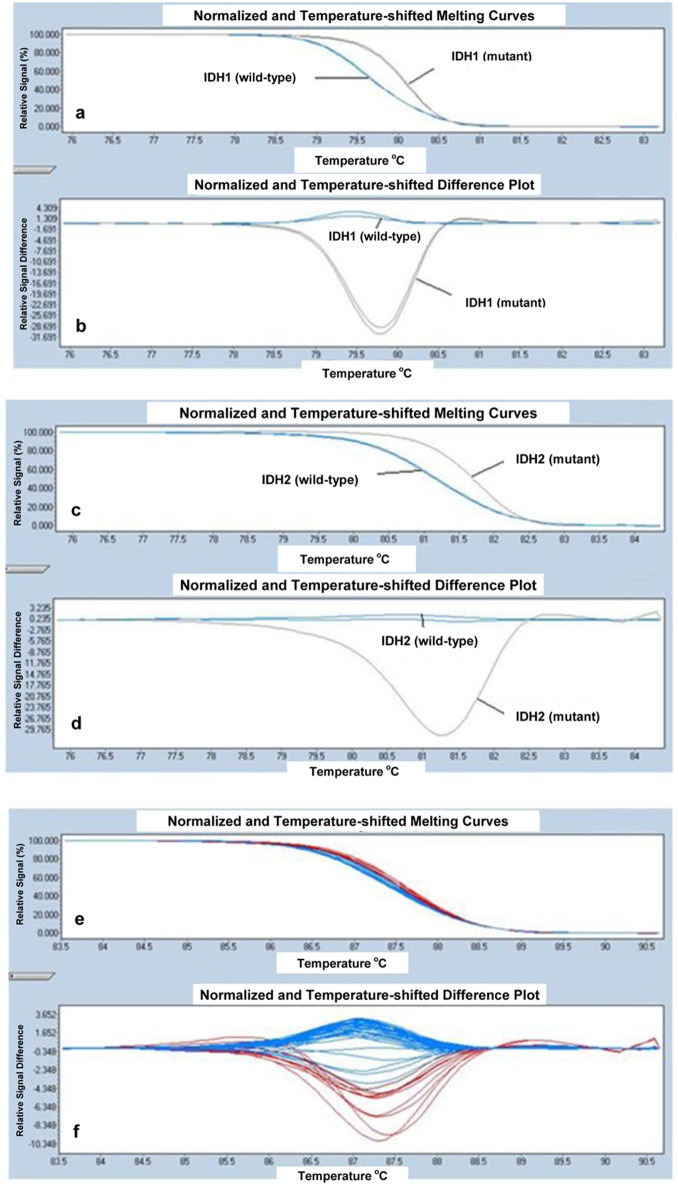
HRM curve analysis for IDH1 (R132) samples. The differential melting properties of wild-type and mutant type IDH1 (R132) using (a) normalized and temperature-shifted melting curves and (b) normalized and temperature-shifted difference plots. HRM curve analysis for IDH2 (R140) samples. The differential melting properties of wild-type and mutant type IDH2 (R140) using (c) normalized and temperature-shifted melting curves and (d) normalized and temperature-shifted difference plots. HRM curve analysis for IDH2 (R172) samples. The melting properties of IDH2 (R172) are shown using (e) normalized and temperature-shifted melting curves and (f) normalized and temperature-shifted difference plots. All samples of IDH2 (R172) showed the same pattern of melting temperature indicating that all patient samples are wild-type for IDH2 (R172).

IDH mutation analysis was performed using high-resolution melting (HRM) in the LightCycler 480 real-time PCR system (Roche Diagnostics), using primers as described below. The primer sequences used to amplify IDH1 exon 4 (R132) were: 5’-CGGTCTTCAGAGAAGCCATT-3’ (forward), and 5’-CACATTATTGCCAACATGAC-3’ (reverse) and IDH2 exon 4 (R140) were: 5’-GCTGAAGAAGATGTGGAA-3’ (forward), and 5’- TGATGGGCTCCCGGAAGA-3’ (reverse), respectively as previously described [11]. IDH2 exon 4 (R172) was amplified using forward, 5’-CCAAGCCCATCACCATTG-3’ and reverse, 5’-CCCAGGTCAGTGGATCCC-3’ [11]. All PCR amplifications were done in a total volume of 20 μL PCR mix containing 10 μL of LightCycler 480 HRM Master Mix, 0.2 μL of each primer, 1.6 μL of MgCl_2_ concentration, 50 ng/μL of genomic DNA and sufficient amount of dH_2_O to complete the volume to 20 μL. PCR mix was dispensed into individual wells of the 96-well plate. The reaction mix was subjected to an initial denaturation of 95 °C for 10 min, followed by 45 cycles of amplification consisting of denaturation at 95 °C for 10 s, annealing at 54 °C for 15 s, and extension at 72 °C for 25 s. Melting was performed with a denaturation step at 95 °C for 1 min, followed by an annealing step at 40 °C for 1 min and a melt from 65 to 95 °C at a ramp rate of 0.02 °C/s. Melting curves from the samples were automatically normalized and analyzed by direct comparison with LightCycler 480 Software, version 1.5.0 SP3 (Roche Diagnostics). All of the patient samples with curves that differed in shape and/or melting temperature were considered as potential variant carriers. The results were confirmed by direct sequence in both directions on ABI 3730 × l DNA analyzers, including PCR primers (Applied Biosystems, Foster City, CA, USA). HRM curve analyses for IDH1 (R132), IDH2 (R140) and IDH2 (R172) are shown in Figure 1a-f respectively.

### Quantification of JAK2V617F allele burden

JAK2 MutaScreen kit (Ipsogen, Luminy Biotech, Marseille, France) was used for the detection of JAK2V617F status and quantitative JAK2V617F allele burden in genomic DNA using TaqMan allelic discrimination [29, 30]. The mutant allele burden was estimated by six-scaled standards of JAK2 V617F mutant allele (2%, 5%, 12.5%, 31%, 50%, and 78%). JAK2V617F mutant allele burdens equal to or less than 50% and greater than 50% were named as low and high JAK2V617F allele burden, respectively.

### Statistical analyses

SPSS version 16 (Prentice Hall, Upper Saddle River, NJ, USA) was used for all stastistical calculations. Numerical variables were summarized as mean (SD). The Chi-square statistics were used to compare categorical variables between the groups. Comparison between categorical and continuous variables was performed using the Mann-Whitney U test (two groups) or Kruskal-Wallis test (more than two groups). The odds ratios (ORs) are accompanied by Cornfield 95% confidence limits (CIs). A two-tailed P value of less than 0.05 was considered significant. Parametric and nonparametric correlation analyses were used to detect associations with IDH mutations. Kaplan-Meier estimates were used to plot OS and LFS in patients with PMF. The curves of OS and LFS were compared by log-rank test.

## Results

### Comparison of patients with ET and PMF according to IDH mutations

IDH (IDH1 or IDH2) mutations were identified in five (6.5%) PMF and two (1.9%) ET patients. Of the five IDH-mutated PMF patients, three displayed the IDH1 R132C mutation, one harbored the IDH1 R132S substitution and one carried the IDH2 R140Q mutation (Table 1). In summary, among IDH-mutated PMF patients, IDH1 and IDH2 mutations were detected in 80% (four in five) and 20% (one in five), respectively. Sixty percent (three in five) of IDH-mutated PMF patients carried the JAK2V617F mutation with the following JAK2V617F allele burdens: 31-50%, 5-12.5% and 31-50%, respectively. Three of 77 PMF patients (3.9%) harbored, simultaneously, IDH and JAK2V617F mutations.

**Table 1:** Clinical and Molecular Characteristics of IDH-Mutated PMF Patients

UPN	Age	G	Karyotype	IDH	JAK2 (allele burden)	FU	OS	Drug	Survival
1*	82	F	Normal	IDH1 R132C	(-)	5	5	Hydroxyurea	Death
2	59	F	Normal	IDH1 R132C	(-)	24	24	Hydroxyurea	Death
3	86	F	Normal	IDH1 R132C	(+) (31-50%)	187	187	Hydroxyurea	Death
4	57	F	Normal	IDH1 R132S	(+) (5-12.5%)	284	284	Hydroxyurea	Alive
5	37	F	Normal	IDH2 R140Q	(+) (31-50%)	4	4	Hydroxyurea	Alive

UPN indicates unique patient number. G: gender; F: female; FU: follow-up (months); OS: overall survival (months). *Patient with leukemic transformation.

Pathogenic alterations in the IDH gene were detected in two of 107 ET cases (1.9%). Of two IDH-mutated ET patients, one displayed the IDH1 R132C substitution and the other showed the IDH2 R140Q mutation (Table 2). The ET patient with the IDH2 R140Q mutation harbored JAK2V617F mutation with an allele burden of 5% while the other patient with IDH1 R132C mutation did not harbor JAK2V617F mutation. One of 107 ET patients (0.9%) cocarried the IDH and JAK2V617F mutations. IDH2 R172 mutations were not detected in the 184 samples studied. Clinical and molecular characteristics of IDH-mutated PMF and ET patients were outlined in Table 1 and 2.

**Table 2:** Clinical and Molecular Characteristics of IDH-Mutated ET Patients

UPN	Age	G	IDH	JAK2 (allele burden)	FU	OS	Drug	Survival
1	55	M	IDH1 R132C	(-)	64	64	Hydroxyurea, ASA	Alive
2	33	F	IDH2 R140Q	(+) (5%)	52	60	Hydroxyurea	Alive

UPN indicates unique patient number. G: gender; F: female; M: male; FU: follow-up (months); OS: overall survival (months).

There was no significant difference in the prevalence of IDH mutation (IDH1 or IDH2) in ET and PMF patients (1.9% and 6.5%, respectively). ET and PMF patients showed no significant difference in the prevelance of combined JAK2V617F and IDH mutations (0.9% and 3.9%, respectively).

### Outcomes of IDH (exon 4) mutations

A total of seven IDH mutations (3.8%) were detected: five in PMF (6.5%) and two in ET patients (1.9%). All alterations in IDH gene were missense mutations. The most common variation was IDH1 R132C, which comprised four of seven IDH mutations (57.1%), followed by IDH2 R140Q (two in seven, 28.6%) and IDH1 R132S (one in seven, 14.3%). Of the seven IDH-mutated patients, five displayed IDH1 (71.4%) and two IDH2 (28.6%) mutations. Among five PMF patients with IDH mutations, four (80%) carried IDH1 mutations (three IDH1 R132C, 60% and one IDH1 R132S, 20%) and one patient harbored IDH2 mutation (one IDH2 R140Q, 20%). One ET patient with IDH mutation harbored IDH1 R132C while the other patient carried IDH2 R140Q substitution.

### ET patients carrying IDH mutations

Two of 107 ET patients (1.9%) displayed IDH mutations. IDH mutations included one IDH1 R132C and one IDH2 R140Q. The ET patient with IDH2 R140Q mutation harbored JAK2V617F mutation with an allele burden of 5% while the other patient with IDH1 R132C mutation did not harbor JAK2V617F mutation. All ET patients carrying IDH mutations were exposed to hydroxyurea treatment and were alive at the end of follow-up. The distribution of IDH mutations in patients with ET is outlined in Table 2.

### Comparison of PMF patients according to the IDH mutation

In 77 consecutive PMF patients, mutant IDH was detected in five patients (6.5%): four IDH1 (three IDH1 R132C and one IDH1 R132S) and one IDH2 (IDH2 R140Q). The distribution of IDH mutations in PMF patients is outlined in Table 1. Three of five IDH-mutated PMF patients (60%) concurrently carried the JAK2V617F mutation. JAK2V617F allele burdens of patients with IDH1 R132C, IDH1 R132S and IDH2 R140Q were 31-50%, 5-12.5% and 31-50%, respectively. All of the IDH mutant PMF patients received hydroxyurea treatment (Table 1).

Clinical and laboratory features of PMF patients according to the IDH mutational status are listed in Table 3.

**Table 3:** Clinical and Laboratory Features of PMF Patients Divided by IDH Mutational Status

PMF	IDH mutant (mean (SD))	IDH wild-type (mean (SD))	P value
Number of patients	5	72	-
Age at recording	64.2 (20)	60.6 (14.2)	0.642
Age at diagnosis	55.4 (20.6)	56.93 (14.1)	0.86
Age at sampling	63.2 (20.1)	59.3 (14.02)	0.679
Females (%)	5 (100%)	38 (52.8%)	0.063
Total leukocyte at diagnosis (mm^3^)	9.662 (5.725)	14.892 (13.886)	0.482
Hb at diagnosis (g/dL)	10 (1.3)	10.6 (2.2)	0.482
HCT at diagnosis (%)	31.4 (4.87)	32.1 (7.13)	0.694
Platelet count at diagnosis (mm^3^)	272.760 (267.777)	444.948 (366.701)	0.193
LDH at diagnosis (U/L)	713 (470)	836 (390)	0.251
Spleen size at diagnosis (mm)	179.4 (30.7)	198.9 (43.7)	0.325
PMF	IDH mutant, n (%)	IDH wild-type, n (%)	P value
Risk factors for cardiovascular diseases	3 (60%)	43 (59.7%)	1
Splenomegaly group	5 (100%)	72 (100%)	0.594
No splenomegaly	0	1 (1.4%)	-
Mild splenomegaly	2 (40%)	15 (20.8%)	-
Massive splenomegaly	3 (60%)	56 (77.8%)	-
Bleeding	3 (60%)	12 (16.7%)	0.048
Need for red blood cell transfusion	1 (20%)	20 (27.8%)	1
Hydroxyurea	5 (100%)	67 (93.1%)	1
History of splenectomy	0	4 (5.6%)	1
AHSCT	0	3 (4.2%)	1
ASA	1 (20%)	46 (63.9%)	0.072
Leukemic transformation	1 (20%)	3 (4.2%)	0.24
Death	3 (60%)	11 (15.3%)	0.039
Thrombosis	1 (20%)	10 (13.9%)	0.548
Thrombosis group	5 (100%)	72 (100%)	0.802
No thrombosis	4 (80%)	62 (86.1%)	-
Arterial	1 (20%)	6 (8.3%)	-
Venous	0	3 (4.2%)	-
Arterial and venous	0	1 (1.4%)	-
JAK2V617F mutation	3 (60%)	55 (76.4%)	0.592
JAK2V617F group	5 (100%)	72 (100%)	0.401
No mutation	2 (40%)	17 (23.6%)	-
Low allele burden	3 (60%)	37 (51.4%)	-
High allele burden	0	18 (25%)	-
Karyotype	5 (100%)	72 (100%)	0.671
Normal	5 (100%)	62 (86.1%)	-
Favorable	0	7 (9.7%)	-
Unfavorable	0	3 (4.2%)	-
DIPSS-plus	5 (100%)	72 (100%)	0.889
Low risk	1 (20%)	14 (19.4%)	-
Intermediate-1 risk	2 (40%)	25 (34.7%)	-
Intermediate-2 risk	2 (40%)	25 (34.7%)	-
High risk	0	8 (11.2%)	-

In PMF, the presence of IDH mutations was not influenced by age at recording, sampling and diagnosis (P = 0.642, P = 0.679 and P = 0.86, respectively). IDH-mutated PMF patients showed a trend towards a higher rate in female patients (100% and 52.8%, respectively; P = 0.063). There were no significant differences in levels of hemoglobin (Hb), hematocrit (HCT), total leukocyte count and platelet count between IDH mutant and wild-type PMF patients. In addition, IDH-mutated PMF patients showed similar levels of LDH with wild-type counterparts.

The mean spleen size and degree of splenomegaly did not differ between IDH-mutated and wild-type PMF patients. Similar results were documented for the two groups with respect to the need for red blood cell transfusion and the presence of risk factors for cardiovascular diseases. The prevalence of total thrombotic events, arterial thrombosis and venous thrombosis did not significantly differ among IDH-mutated and wild-type PMF patients (P = 0.548, P = 0.662 and P = 0.558, respectively).

IDH mutant PMF patients demonstrated higher prevalence of bleeding complications than IDH wild-type PMF patients (60% and 16.7%, respectively; P = 0.048). As regards the localization of bleeding events among PMF patients, 20% of IDH mutant PMF patients experienced gastrointestinal bleeding and 20% intracranial hemorrhage, while gastrointestinal hemorrhage occurred in 5.6% and intracranial hemorrhage in 1.4% of IDH wild-type PMF patients (P = 0.061). There was a trend towards severe bleeding in IDH-mutated PMF patients compared to wild-type PMF patients (P = 0.061). A trend towards a lower prevalance of acetylsalicylic acid (ASA) use was present in the IDH mutant PMF patients compared to wild-type counterparts (20% and 63.9%, respectively; P = 0.072). This can be explained by the increased frequency of bleeding complications in IDH-mutated PMF patients.

No significant difference was observed in the frequency of JAK2V617F mutation between IDH-mutated and wild-type PMF patients (60% and 76.4%, respectively; P = 0.592). Similarly, the quantitative JAK2V617F allele burdens showed no difference among PMF patients with or without IDH mutation (P = 0.704). Likewise, the prevalence of JAK2V617F-positive patients with mutant allele burden in upper quartile ranges was not different in IDH mutant PMF patients compared to IDH wild-type PMF patients (0 and 25% respectively; P = 0.401). Three of 58 (5.2%) JAK2V617F-positive and two of 19 (10.5%) JAK2V617F-negative PMF patients displayed IDH mutations (exon 4) (P = 0.36). Similarly, JAK2V617F-positive and JAK2V617F-negative PMF patients showed no significant difference for the frequency of IDH1 and IDH2 mutations (3.4% and 10.5%, respectively; P = 0.253 and 1.7% and 0, respectively; P = 1). As regards the JAK2V617F allele burden, the prevalence of IDH mutation did not differ across all three groups: 10.5% (two of 19) in JAK2V617F wild-type, 7.5% (three of 40) in patients with low JAK2V617F allele burden and 0 in patients with high JAK2V617F allele burden (P = 0.401). The prevalence of IDH1 mutations was 10.5% in JAK2V617F wild-type PMF patients, 5% in PMF patients with low JAK2 allele burden and 0 in PMF patients with high JAK2 allele burden while 0 of JAK2V617F wild-type PMF patients, 2.5% of PMF patients with low JAK2 allele burden and 0 of PMF patients with high JAK2 allele burden displayed IDH2 mutations (P = 0.352 and P = 0.626, respectively). With respect to IDH mutation status, PMF patients showed no significant difference in the prevalence of hydroxyurea use, use of other medical treatments, rate of AHSCT and history of splenectomy (P > 0.05).

DIPSS-plus risk stratification was similar for PMF patients with and without IDH mutations (P = 0.889). Among IDH-mutated and wild-type PMF patients, the distribution of karyotype abnormalities was not different (P = 0.671). The prevalence of normal karyotype, favorable and unfavorable karyotype abnormalities in IDH mutant and wild-type PMF patients were as follows: 100%, 0 and 0; 86.1%, 9.7% and 4.2%, respectively (P = 0.671).

Higher rates of death were documented in IDH-mutated PMF patients compared to wild-type PMF patients (60% and 15.3%, respectively; P = 0.039). The rate of leukemic transformation was higher in IDH-mutated PMF patients than wild-type counterparts but with no statistical significance (20% and 4.2%, respectively; P = 0.24).

In PMF patients, the presence of IDH mutation did not correlate with HCT level, total leukocyte count, platelet count, LDH level, mean spleen size, total thrombotic events, arterial thrombosis and venous thrombosis (r < 0.2). There was a weak positive correlation between IDH mutation and bleeding events (r = 0.27). In PMF, the copresence of JAK2V617F and IDH mutations did not correlate with HCT level, total leukocyte count, platelet count, LDH level, bleeding complications, total thrombotic events, arterial thrombosis, venous thrombosis and death (r < 0.2).

### Kaplan-Meier survival curves in PMF patients

**Figure 2:**
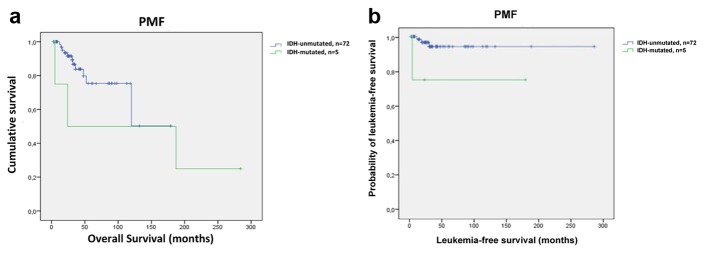
Survival outcomes and leukemia-free survival in PMF patients (n = 77). (a) Kaplan-Meier survival analysis of PMF patients with respect to IDH mutation status. OS was similar between patients with IDH mutations and those with no mutations (mean 125 months; 95% CI: 11 - 238 and 128 months; 95% CI: 98 - 157, respectively; P = 0.351). (b) Leukemia-free survival data for 77 PMF patients stratified by the status of IDH mutations. PMF patients with IDH mutations had shorter LFS compared to patients without IDH mutations (mean 169 months; 95% CI: 159 - 180 and 214 months; 95% CI: 95 - 332, respectively; P = 0.024).

Although the number of IDH-mutated PMF patients was small, Kaplan-Meier plots were used to calculate OS and LFS. OS did not differ between IDH mutant and wild-type PMF patients (mean 125 months; 95% CI: 11 - 238 and 128 months; 95% CI: 98 - 157, respectively; P = 0.351) (Fig. 2a). Univariate analysis revealed significantly shorter LFS in IDH-mutated PMF patients compared to IDH wild-type PMF patients (mean 169 months; 95% CI: 159 - 180 and 214 months; 95% CI: 95 - 332, respectively; P = 0.024) (Fig. 2b).

## Discussion

IDH1 and IDH2 catalyze an essential step in the Krebs cycle that converts isocitrate to α-KG. Mutant IDH not only loses their normal activity in catalyzing NADP+-dependent oxidative decarboxylation of isocitrate into α-KG, but also gains a new function: catalyzing the α-KG to 2-HG (2-4). 2-HG is found elevated in the serum of patients with gliomas and AML harboring IDH mutations, suggesting that 2-HG could be a biomarker for IDH-mutated glioma and AML [3, 5].

Although many investigators performed mutational analysis of large AML cohorts, limited number of studies previously assessed the prevalence of IDH1 and IDH2 mutations in Ph-negative MPNs [1, 10, 11, 15, 24]. IDH mutations have also been identified in AML patients transformed from MPNs [10, 15, 16]. In one of these studies, mutant IDH was detected in five of 16 post-MPN AML patients (31%) whereas no mutations were detected in 180 patients with chronic-phase PV and ET [16]. Abdel-Wahab et al identified IDH1 mutations in 9.5% of post-MPN AML patients [10]. In another study, 166 patients with chronic-phase MPN (77 PMF, two post-PV myelofibrosis, two post-ET myelofibrosis, 47 ET, and 38 PV) and 34 blast-phase MPN were screened for IDH1 and IDH2 mutations [15]. Nine IDH mutations including five IDH1 (four R132C and one R132S) and four IDH2 (three R140Q and one R140W) were observed. The frequencies of IDH mutations for blast-phase MPN and PMF were 21% and 4%, respectively. IDH mutations were not detected in ET or PV. The results of the aferomentioned study implied that IDH mutations are relatively frequent in blast-phase MPN and rare in chronic-phase MPN. Also, among Ph-negative MPNs, these mutations were observed to be limited to PMF [15]. In a review by Shih et al, the mutational frequencies of IDH1 and IDH2 mutations were 2.5-5% for MPN patients [31]. In the same report, IDH mutations were described in 1-2% of PV, 1-2% of ET and 2-5% of PMF patients [31]. Brecqueville et al found two IDH2 mutations in 14 blast-phase MPN patients but no IDH mutations in 135 chronic-phase MPN patients [32]. In a study by Pardanani et al, a total of nine IDH mutations in 78 chronic-phase MPN patients (12%) were reported including four in 25 PMF (16%), four in 39 PV (10%) and one in 14 ET patients (7%) [33]. In another study, IDH mutations were not detected in 62 chronic-phase MPN patients (52 ET, five PMF and five PV) [34]. The largest study that investigated the frequency of IDH1 and IDH2 mutations in Ph-negative MPN patients included 594 ET, 421 PV, 312 PMF, 95 post-PV/ET myelofibrosis and 51 blast-phase MPN patients [11]. A total of 38 IDH mutations (19 IDH2 R140, 18 IDH1 R132 and one IDH2-R172) were described: five ET (0.8%), eight PV (1.9%), 13 PMF (4.2%), one post-PV/ET myelofibrosis (1%) and 11 blast-phase MPN (21.6%) [11]. Consequently, in that study, mutant IDH was documented to be more frequent in blast-phase MPN than chronic-phase MPNs [11]. Two other studies also investigated IDH mutations in a large cohort of PMF patients [1, 24]. In one study, the frequency of IDH mutations in PMF patients was 4% (12 in 301) and in the other study, the prevalences of IDH1 and IDH2 mutations in PMF patients from Mayo Clinic were 3% and 2%, respectively [1, 24]. In the latter study, the prevalence of IDH1/IDH2 mutations in PMF patients from the European cohort was 2.6% [24]. In summary, several studies have examined the prevalence of IDH mutations in chronic-phase MPNs [1, 11, 15, 16, 24, 32-34]. The aforementioned limited number of studies reported variable prevalences for IDH mutations in Ph-negative MPNs. There are no previous studies that assessed IDH mutations in Ph-negative MPNs in Turkey. In this study, we genotyped a cohort of 184 Turkish Ph-negative MPNs (107 patients with ET and 77 with PMF) for mutations of IDH1 and IDH2 at Molecular Hematology Laboratory of Istanbul University. The mean ages of ET (54.2% females) and PMF (55.8% females) were 56.3 years (SD 14.5) and 60.8 years (SD 14.5), respectively. Of 77 PMF patients, five (6.5%) had IDH mutations, compared to two of 107 ET patients (1.9%); however, this difference was not statistically significant.

Several studies reported that HRM curve analysis is as reliable as direct nucleotide sequencing for assessing IDH mutations in AML and MPN patients [15, 35]. Also, it is acknowledged that HRM is a rapid, practical and cost-effective screening method to detect IDH mutation. On the basis of these findings, we used HRM curve analysis to detect IDH1/IDH2 mutations in our cohort of Ph-negative MPN patients. The mutant IDH was detected in 1.9% of our chronic-phase ET and 6.5% of PMF patients. In line with a previous study, our findings suggested that both IDH1 and IDH2 mutations can occur in chronic-phase ET and PMF patients, yet at low frequency [11]. Our results indicate that IDH mutations can occur early in MPN development. To date, several studies demonstrated that IDH mutations were more prevalent in blast-phase MPN compared to chronic-phase MPN [10, 15, 16]. These observations favor the possibility that IDH mutations in MPN are early genetic triggers of leukemic transformation supporting the model by Yan et al, which suggested IDH mutations occur early during gliomagenesis and provide progression from low-grade or anaplastic astrocytomas to secondary glioblastomas [7].

Candidate gene resequencing of IDH1 and IDH2 in a very large cohort of MPN patients demonstrated that mutations in IDH1/2 occur most commonly at the IDH2 R140 residue in MPNs, followed by IDH1 R132 and IDH2 R172 [11]. The same order of frequency for the aforementioned IDH variants was reported in another study evaluating IDH mutations among chronic-phase PMF patients [1]. The mutant IDH was detected in seven of our 184 Ph-negative MPN patients (3.8%). The following IDH variants were displayed: four IDH1 R132C, 57.1%, two IDH2 R140Q, 28.6% and one IDH1 R132S, 14.3%. Consequently, the frequencies of IDH1 and IDH2 mutations in the seven patients with IDH mutations were 71.4% (five in seven) and 28.6% (two in seven), respectively. Of the five PMF patients with IDH mutations, four (80%) carried IDH1 mutations (three IDH1 R132C, 60% and one IDH1 R132S, 20%) and one patient harbored IDH2 mutation (one IDH2 R140Q, 20%). One IDH-mutated ET patient harbored IDH1 R132C, whereas the other patient carried IDH2 R140Q substitution. In our cohort, no mutation involving residue R172 of IDH2 gene was detected which was consistent with some studies of AML, chronic-phase and blast-phase MPN patients [8, 15, 16]. Contrary to the previous studies in our 184 Ph-negative MPNs, the most common variant of IDH mutations was IDH1 R132, followed by IDH2 R140 [1, 11].

A limited number of studies have investigated the association of gender and age with IDH mutational status in Ph-negative MPNs [1, 11]. In one study, the presence of IDH mutations in chronic and blast-phase PMF was reported not to be influenced by gender or age in contrast to the findings of another series of 301 PMF patients where IDH mutation was associated with older age [1, 11]. However, in the last study, PMF patients did not differ in gender with respect to IDH mutation status [1]. We did not detect any significant difference in age between IDH mutant and wild-type PMF patients. Also, our IDH-mutated PMF patients showed a trend towards a higher rate in female patients.

To the best of our knowledge, only one previous study had compared the clinical and laboratory characteristics of IDH mutant PMF patients with those of wild-type counterparts [1]. Consistent with the findings of that study, levels of Hb, HCT, leukocyte and platelet counts did not differ between our IDH mutant and wild-type PMF patients [1]. Tefferi et al reported that the presence of palpable spleen size of greater than 10 cm did not differ between IDH-mutated and -unmutated PMF patients [1]. Likewise, in our study, mean spleen size and the degree of splenomegaly did not differ between IDH-mutated and wild-type PMF patients.

To our knowledge, no previous study of MPNs has assessed the relationship of IDH mutation with thrombosis, bleeding events and LDH levels. In our study, IDH mutant and IDH wild-type PMF patients showed no significant difference with respect to LDH levels, rate of total thrombotic events, arterial thrombosis and venous thrombosis. However, the prevalence of bleeding complications was significantly higher in our IDH mutant PMF patients compared to wild-type PMF patients (60% and 16.7%, respectively). Also, in our PMF patients, a weak positive correlation was found between IDH mutation and bleeding complications. As regards the localization of bleeding complications among PMF patients, 20% of IDH mutant PMF patients experienced gastrointestinal bleeding and 20% intracranial hemorrhage, whereas gastrointestinal hemorrhage occurred in 5.6% and intracranial hemorrhage in 1.4% of IDH wild-type PMF patients. Consequently, there was a trend towards severe bleeding in IDH mutant PMF patients compared to wild-type PMF patients.

In a series of 200 MPN patients, IDH mutations were reported at a frequency of 21% in blast-phase MPN and 4% in PMF [15]. In that study, three of the nine IDH mutant MPN patients (33.3%) harbored JAK2V617F mutation [15]. One previous study reported that the frequency of IDH mutations did not differ between JAK2V617F mutant and JAK2V617F wild-type MPN patients (3.6% and 4.2%, respectively) [11]. In line with this observation, our JAK2V617F-positive and -negative ET patients showed similar incidences for the IDH mutation (1.6% and 2.3%, respectively). In addition, there was no difference in the prevalences of the IDH mutation between our PMF patients with and without JAK2V617F mutation (5.2% and 10.5%, respectively). In another study, mutant IDH was detected in 12 of 301 PMF patients (4%) and six of 12 IDH-mutated PMF patients (50%) also harbored JAK2V617F mutation [1]. We did not find any significant difference in the incidence of JAK2V617F mutation between IDH mutant and wild-type PMF patients (60% and 76.4%, respectively). Moreover in our patients, JAK2V617F mutant allele load showed no difference between our PMF patients with and without IDH mutation. Furtheron, the prevalence of IDH mutation did not differ when our ET and PMF patients were stratified according to JAK2V617F allele burden separately. In our study, 40% of IDH-mutated PMF patients did not co-express JAK2V617F in line with the study of Pardanani et al which suggested that IDH mutations emerge independently of JAK2V617F mutation [15]. In a series of 301 consecutive patients with PMF, JAK2V617F allele burdens in the six patients co-carrying mutant IDH were 1%, 7%, 22%, 27%, 30% and 96%, respectively [1]. In our study, three of the five IDH mutant PMF patients (60%) also expressed JAK2V617F mutation and JAK2V617F allele burdens in these three patients were 31-50%, 5-12.5% and 31-50%, respectively. We detected the mutant IDH in two of 107 ET patients (1.9%). One of these ET patients displayed JAK2V617F mutation with an allele burden of 5% while the other patient with IDH1 R132C mutation did not display JAK2V617F mutation.

A limited number of studies have assessed the cytogenetic risk categories of IDH mutant MPN patients [1, 11, 24]. Tefferi et al reported that cytogenetic findings in IDH mutant chronic-phase PMF patients often corresponded to a low or intermediate risk category yet with no statistical significant difference [11]. In the same study, presence of complex karyotype was found to be less frequent in IDH mutant blast-phase PMF as opposed to wild-type counterparts [11]. In another study, IDH mutant and wild-type PMF patients showed no difference with respect to cytogenetic risk categories [1]. In a study which included 279 PMF patients from Mayo Clinic, the frequencies of IDH mutations were similar between PMF patients with normal and abnormal cytogenetic profiles [24]. In our study, all of the five IDH mutant PMF patients displayed normal karyotype. Yet, no difference was observed between our IDH-mutated and wild-type PMF patients with respect to the distribution of karyotypic categories. Only one previous study had investigated the relationship of IDH mutation with DIPSS-plus risk stratification in PMF patients [1]. In that study, DIPSS-plus risk distribution was similar between IDH mutant and IDH wild-type PMF patients [1]. Consistent with this finding, we did not find any significant difference in DIPSS-plus risk stratification among PMF patients with and without IDH mutations [1]. Excluding AML patients, a limited number of studies have investigated the prognostic impact of IDH mutations in chronic myeloid disorders, including MDS and MPN [1, 11, 12, 24]. In one of these studies, the presence of IDH mutations had adverse effect on survival in both blast-phase PMF and blast-phase MPN but not in chronic-phase PMF [11]. However, that particular study included only 111 chronic-phase PMF patients with complete clinical information and hence was not a thorough prognostic analysis [1]. In another series of 301 chronic-phase PMF patients, multivariate analysis showed worse survival and inferior LFS in the presence of IDH mutation [1]. That study suggested that IDH mutations predict leukemic transformation in PMF patients and increase the likelihood of leukemogenic collaboration with JAK2V617F [1]. The largest study examining the impact of IDH mutations in PMF patients included 279 patients from Mayo Clinic and 483 patients from the European cohort [24]. In the European cohort, IDH1 or IDH2 mutations predicted leukemic transformation but were not associated with inferior OS. However, in the Mayo cohort, univariate analysis identified that IDH1 mutations were associated with worse OS. Furtheron, in the same cohort, both univariate and multivariate analyses identified IDH1 mutations as significant prognostic factors for LFS [24]. In our study, higher rates of death were documented in IDH-mutated PMF patients compared to wild-type counterparts (60% and 15.3%, respectively). However, OS did not differ between our IDH mutant (n = 5) and wild-type PMF patients (n = 72). The rate of leukemic transformation was higher in our IDH-mutated PMF patients than wild-type counterparts but with no statistical significance (20% and 4.2%, respectively). Although the number of IDH-mutated PMF patients was small (n = 5), we observed a significantly shorter LFS in IDH-mutated PMF patients compared to IDH wild-type PMF patients (n = 72). Consequently in our study group, parallel to the findings by European cohort of the study by Vannucchi et al, LFS was predicted by IDH mutations whereas IDH-mutated PMF patients did not show inferior OS compared to IDH-wild-type PMF patients [24].

In our study group, all IDH mutations detected in PMF patients were associated with normal karyotype yet predicted inferior LFS. This finding suggests that IDH mutations may represent an independent prognostic biomarker in PMF.

Abdel-Wahab et al reported that DH1 mutations were most commonly observed in MPN/AML patients with wild-type JAK2V617F [10]. The frequency of IDH1 mutation was higher in our JAK2V617F wild-type PMF patients compared to JAK2V617F-mutated PMF patients yet the difference was not statistically significant (10.5% and 3.4%, respectively). Our PMF and ET patients showed no significant difference in the copresence of JAK2V617F and IDH mutations (three in 77, 3.9% and one in 107, 0.9%, respectively). In PMF patients, the presence of combined JAK2V617F and IDH mutations did not correlate with HCT level, leukocyte count, platelet count, LDH level, bleeding complications, total thrombotic events, arterial thrombosis, venous thrombosis and death. Further investigations are required to determine the impact of copresence of JAK2V617F and IDH mutations on disease course and complications in Ph-negative MPNs.

The incidence of IDH mutation was higher in our PMF patients (five in 77, 6.5%) than ET patients (two in 107, 1.9%), yet the difference did not reach statistical significance. During follow-up, one of the five IDH mutant PMF patients (20%) underwent leukemic transformation. Our findings support the possibility that IDH mutations are early genetic events in Ph-negative MPN patients and increase leukemic transformation in PMF. Thus, it can be inferred that IDH mutations may play a role in prediction of leukemic transformation in PMF patients.

In our study, approximately 2.3% (one in 43) of JAK2V617F-negative ET patients harbored the IDH mutations. The frequency of IDH mutations in our JAK2V617F-negative PMF patients was 10.5% (two in 19). Based on our findings, it may be deduced that analysis of the IDH genes provided an additional 2.3% increase in ET and a 10.5% increase in PMF patients with a detected molecular marker of clonality.

In conclusion, IDH mutations had significant impact on LFS in PMF patients. Also, IDH mutations were associated with high rate of death but did not influence OS in PMF. Despite the limited number of IDH-mutated PMF patients included, our observations support that IDH mutations provide genetic events in the pathogenesis and prognosis of PMF patients. Clearly, screening for IDH mutation might be added to future clinical trials and prospective observational studies in PMF. Given the small number of IDH-mutated ET patients in our study group, we could not determine the impact of these genetic alterations on patient characteristics. Larger number of ET patients are needed to detect the prognostic importance of IDH mutations in ET patients.
